# The Effect of Polyhydroxy Fullerene Derivative on Human Myeloid Leukemia K562 Cells

**DOI:** 10.3390/ma15041349

**Published:** 2022-02-11

**Authors:** Wei Guo, Xing Liu, Lianjie Ye, Jie Liu, Kollie Larwubah, Ge Meng, Weiqiang Shen, Xiangxian Ying, Jun Zhu, Shengjie Yang, Jianjun Guo, Yanrong Jia, Meilan Yu

**Affiliations:** 1 College of Life Science and Medicine, Zhejiang Sci-Tech University, Hangzhou 310018, China; gw980310@163.com (W.G.); xingliu6699@163.com (X.L.); 17865933628@163.com (L.Y.); kaki0925@163.com (J.L.); larwubahkollie@gmail.com (K.L.); ziminpigeon@163.com (G.M.); 15524353118@sohu.com (W.S.); biojjguo@zstu.edu.cn (J.G.); jiayr102528@163.com (Y.J.); 2 Shaoxing Academy of Biomedicine, Zhejiang Sci-Tech University, Shaoxing 312030, China; yangshengjie@wahaha.com.cn; 3 College of Biological and Environmental Engineering, Zhejiang University of Technology, Hangzhou 310014, China; yingxx@zjut.edu.cn; 4 Hangzhou Wahaha Co., Ltd., Hangzhou 310018, China; zhujun13677@wahaha.com.cn

**Keywords:** fullerenol, myeloid leukemia, proliferation, apoptosis

## Abstract

The use of nanomedicines for cancer treatment has been widespread. Fullerenes have significant effects in the treatment of solid tumors. Here, we are going to study the effects of hydroxylated fullerene C_60_(OH)_n_(n = 18–22) treatment on chronic myeloid leukemia cell proliferation and investigate its toxicity. The results showed that hydroxylated fullerene C_60_(OH)_n_ (n = 18–22) at low concentrations (less than 120 μM) not only had apparent toxic side effects, but also promoted the growth of K562 cells, while a high concentration of C_60_(OH)_n_ had different degrees of inhibition on K562 cells. When the concentration is higher than 160 μM, the K562 cells showed morphological changes, the mitochondrial membrane potential decreased, the cell cycle was blocked in the stage of G2-phase, and cell apoptosis occurred, which may cause apoptosis, autophagy, and a variety of other damage leading to cell death. Meanwhile, it also indicated that its inhibition of solid tumors might be related to the tumor microenvironment; we verified the safety of fullerene without apparent cellular toxicity at a specific concentration.

## 1. Introduction

Since its discovery in 1985, fullerene has received much interest and been under investigation as a carbon nanomaterial [1]. Due to the water-insolubility of unmodified fullerenes, various physical and chemical modifications have been used to improve the primordial fullerene so that it has superb water-solubility and biocompatibility [2,3,4,5]. After the modification, its unique structure and physicochemical properties give it a wide range of applications in biomedicine and other domains [6,7,8,9,10,11,12,13,14]. Polyhydroxylated fullerenes (known as fullerenols) are the most commonly utilized because of their excellent targeting, slow-release properties, long-term effects, less dosage, and fewer adverse reactions, and so on [15]. Lajos P et al. [8] comprehensive analysis shows that Gd@C_82_(OH)_22_ NPs maintain their remarkable anti-cancer effect in a variety of solid tumors. Using the luciferase-expressing mouse breast cancer cell line 4T1-luc as a xenograft model, Chunying Shu et al. [16] discovered that activated Gd-Ala could make full use of oxygen in blood vessels to create ROS, resulting in partial or complete vascular disruption. These obtained results clarify that fullerene derivative nanomaterials are potential candidates for cancer drugs with high efficacy and low toxicity [8,17], which may open a new perspective for biomedicine. [5] demonstrated the superiority of C_70_-Lys compared with C_70_-Ala against the chemotherapy injuries that induced by doxorubicin. Chunru Wang et al. [5] found that C70-Lys is superior to C70-Ala in terms of doxorubicin-induced chemotherapeutic damage. Many scientists such as Yuliang Zhao et al. [18] believe that the anti-tumor effect of fullerenes is mainly through changing the tumor microenvironment. Mingming Zhen et al. [19] demonstrated that GF-Ala nanoparticles reprogram TAMs from tumor promoting M2 phenotype to tumoricidal M1 phenotype and increase the infiltration of cytotoxic T lymphocytes, rebuilding the immunosuppressive tumor microenvironment and achieving effective inhibition of tumor growth. Zhiyi Chen [20] found that in the acidic tumor microenvironment, the hydrophobic-to-hydrophilic conversion of the pH-responsive polymer DOX-RNPs leads to drug release simultaneously, and no noticeable histological changes were observed in major organs of mice treated with RNPs. Therefore, it is necessary to study the biological characteristics and clinical application of polyhydroxy-fullerenols in biomedicine, especially in malignant tumors and non-solid tumors.

Reactive Oxygen Species (ROS) are a group of oxygen-containing free radicals and free-radical-forming Peroxide related to oxygen metabolism in living organisms. ROS have positive physiological effects in a certain space, time and limit, but excessive ROS can lead to oxidative stress and metabolic disorder, which can lead to various diseases including tumor [21,22]. Fullerene, known as a “radical sponge” [23], can react readily with free radicals as a detoxifying system to effectively quench active radicals or suppress ROS to lower free-radical-induced detrimental effects and maintain ROS in biological systems [24].

The most common non-solid tumor is known as leukemia; leukemia is a clonal malignant disease characterized by aberrant hematopoietic stem cells [25]. Chemotherapy is currently the mainstream for clinical Leukemia treatments. Chronic myeloid leukemia (CML) is a kind of hematological malignancy characterized by the expression of oncogenic kinase BCR-ABL. Since the K562 protocell is a malignant hematopoietic cell with various differentiation potentials that is a susceptible target for natural killer cells in vitro and is widely employed in this study, we picked it as a non-solid tumor research target. Over the last few years, there has been a significant shift in CML treatment. In western countries, the typical age of CML patients is about 57 years, more than 20% of patients are over 70 years old and 5% of patients are children and adolescents [26]. Still, the median age at diagnosis in Asia and Africa is 50 years [27]. Compared with other tumors, the harm to humans is more obvious and prominent. Finding leukemia treatment medications with high efficacy and minimal toxicity is a pressing issue [28]. 

However, throughout these studies on the effect of fullerenes on cancer—most of them focused on the effect of fullerenes in solid tumors [16,19]—there is little research on the specific non-solid tumor cancer: blood cancer. Some research on the anticancer efficacy of C60(OH)_n_ (n = 18–22) in vitro on the chronic myeloid leukemia cell line K562 cells has been reported. Investigating the proliferation and toxicity of fullerenes on myeloid leukemia cells will not only help to explore the mechanism of blood malignant tumor suppression, but also may provide more references and options for further development of fullerenol as a target non-tumor therapeutic.

## 2. Materials and Experiment Procedures

### 2.1. Materials

Water-soluble polyhydroxy fullerenol C_60_(OH)_n_ (n = 18–22) was synthesized in an aqueous phase by Xiamen Funaxin Materials Technology Co., Ltd., Xiamen, China. The fetal bovine serum was purchased from Zhejiang Sijiqing Biotechnology Co., Ltd., Hangzhou, China. Doxorubicin hydrochloride was purchased from Shanghai McLean biochemical technology Co., Ltd., Shanghai, China. Cell Counting kit-8 was purchased from Tongren Institute of Chemistry, Japan. Cell Cycle Detection Kit, Apoptosis Detection Kit, and Jc-1 Cell Apoptosis Mitochondrial Membrane Potential Detection Kits were purchased from Nanjing Kaiji Biotechnology Development Co., Ltd., Nanjing, China. 

Human chronic myelocytic leukemia cell line K562 derived from the Institutes of Biological Sciences, Chinese Academy of Sciences, Shanghai, China. Even though K562 can be considered as a model system for myelogenous leukemia, it is a transformed/immortalized cell line; therefore, all experimental results are focused on this cell line.

### 2.2. Cell Proliferation Inhibition Assay

Leukemia cells K562 and normal liver cells L-02 in the logarithmic phase were inoculated on a 96-well culture plate with a density of 4 × 10^4^ cells/mL. The control group was added with the same amounts of cells and a complete medium of RPMI-1640. Fullerenol C_60_(OH)_n_ (n = 18–22) diluted with RPMI-1640 culture medium was added to the treatment group to obtain final concentrations of 40, 60, 80, 100, 120, 160, 240, and 320 μmol/L, respectively. Adriamycin was used as the positive control group and blank holes were set. Six multiple wells were set for each well, the A450 values of each well at 24 and 48 h were determined by enzyme-linked immunoassay, and the inhibition rate of cell proliferation was calculated according to the following formula:

Proliferation inhibition rate = (A control − A administration)/(A control − A blank) × 100%

### 2.3. Cell Cycle Assay

K562 cells in the logarithmic phase were adjusted to the concentration of 1.5 × 10^5^ cells/mL, and the cells were inoculated into a 6-well culture plate, 2 mL of cell fluid was added into each well, the experimental group immediately added C_60_(OH)_n_ (n = 18–22) with the final concentration of 0, 40, 60, 80, 100, 120, 160, 240 and 320 μmol/L, respectively. The control added the same amounts of cells and complete medium of RPMI-1640. The cells were cultured at 37 °C and 5% CO_2_ for 48 h and then collected. Pre-cooled PBS was rinsed 2 times, and the cells were resuspended in the pre-cooled 75% ethanol for more than 24 h at −20 °C. Before staining, they were washed with PBS and the cell density was adjusted to 1 × 10^6^ cells/mL. Then the cells were collected in the flow tube, added 100 μL of RNase A, bathed in the water at 37 °C for 30 min, then add 400 μL PI staining solution, slowly and fully resuspended cells. The cells were incubated at 4 °C in dark for 30 min, filtered with 400 nylon membranes, then placed in an ice bath and flow cytometry was used to detect the red fluorescence and light scattering at the excitation wavelength of 488 nm, the changes of the cell cycle were analyzed. The cell proliferation index was calculated according to the formula:

PI = (S + G2/M)/(G0/G1 + S + G2/M)

### 2.4. Cell Morphology Assay

The growth inhibition effect of different concentrations of C_60_(OH)_n_ (n = 18–22) on human leukemia cell line K562 in vitro for 48 h was observed by light microscopy. K562 cells were inoculated in a 96-well plate at a concentration of 1 × 10^4^ cells/well, treated with C_60_(OH)_n_ at different doses for 48 h, then using fluorescence microscope observation and photography, the growth inhibition of K562 cells by C_60_(OH)_n_ was observed.

### 2.5. Detection of Apoptosis

The grouping and dosing status of the K562 cells were the same as that of the cell cycle assay. The blank control group and the monochrome control group were set and cultured at 37 °C and 5% CO_2_ for 48 h. About 1 × 10^6^ cells were collected by centrifugation and washed with precooled PBS for 2 times, PBS was discarded and 100 μL binding buffer was added to make a single-cell suspension. Before staining, the cells were filtered with a 400-mesh sieve for 2 times, Annexin V-FITC 5 μL and PI 5 μL were added in turn, after mixing stained in the dark at 4 °C for 30 min, the apoptosis rate of each group was detected by flow cytometry within 1 h after 400 μL binding buffer was added.

### 2.6. Induction of Apoptosis and AO/EB Double Staining

After the cells reached 80% confluence and C_60_(OH)_n_ was added for 48 h, the single-cell suspension was prepared. The cell concentration was adjusted to 1 × 10^6^ cells/mL with PBS, 25 μL cell suspension was taken, then the cells were treated with 2 μL of AO (100 μg/mL) and 2 μL EB (100 μg/mL), beaten and mixed, dropped on the slide, covered and the slide sealed, and the morphology of apoptotic cells by fluorescence microscope was observed.

### 2.7. Analysis of Mitochondrial Membrane Potential

The cell suspension was washed with JC-1 staining buffer solution for 2 times, the cell concentration was adjusted to 1 × 10^6^ cells/mL, 0.5 mL of JC-1 staining solution with a final concentration of 10 μg/mL was added, and the mixture was then placed in a CO_2_ incubator for further incubation in the dark for 20 min. Centrifuge with 900× *g* for 6 min, discard supernatant, wash with JC-1 staining buffer solution twice, centrifuged with 900× *g* for 6 min every time. Discard the supernatants and add 500 μL JC-1 stain buffer per tube. The cells were detected by flow cytometry, the detection data were obtained and analyzed by Cell Quest functional software. The excitation wavelength was 488 nm, and 10,000 cells were collected for each sample.

### 2.8. Electron Microscopy of Cell Apoptosis

K562 cells in the logarithmic growth stage were inoculated in a 6-well culture plate with 1.5 × 10^8^ cells/mL, and different concentrations of C_60_(OH)_n_ were added for intervention. The control group was supplemented with an equal volume of serum-containing medium without drugs. After culturing for 48 h, 800 r/min was centrifuged for 10 min. The culture medium was discarded and 2.5% glutaraldehyde was added to fix it for 2 h. Samples were prepared according to the requirements of the transmission electron microscope experiment, including embedding, polymerization, and ultra-thin slicing machine. After double staining with lead citrate, the ultrastructural characteristics of the cells were observed under the electron microscope.

### 2.9. Data Analysis

All experiments were repeated independently at least three times and all data presented are means or means ± standard deviations. All analyses were made using SPSS 11.5 statistical software package and *p* < 0.05 were recognized as statistically significant (*) and *p* < 0.01 were greatly statistically significant (**).

## 3. Results

### 3.1. Effects of C_60_(OH)_n_ on the Proliferation of K562 Cells

**Figure 1:**
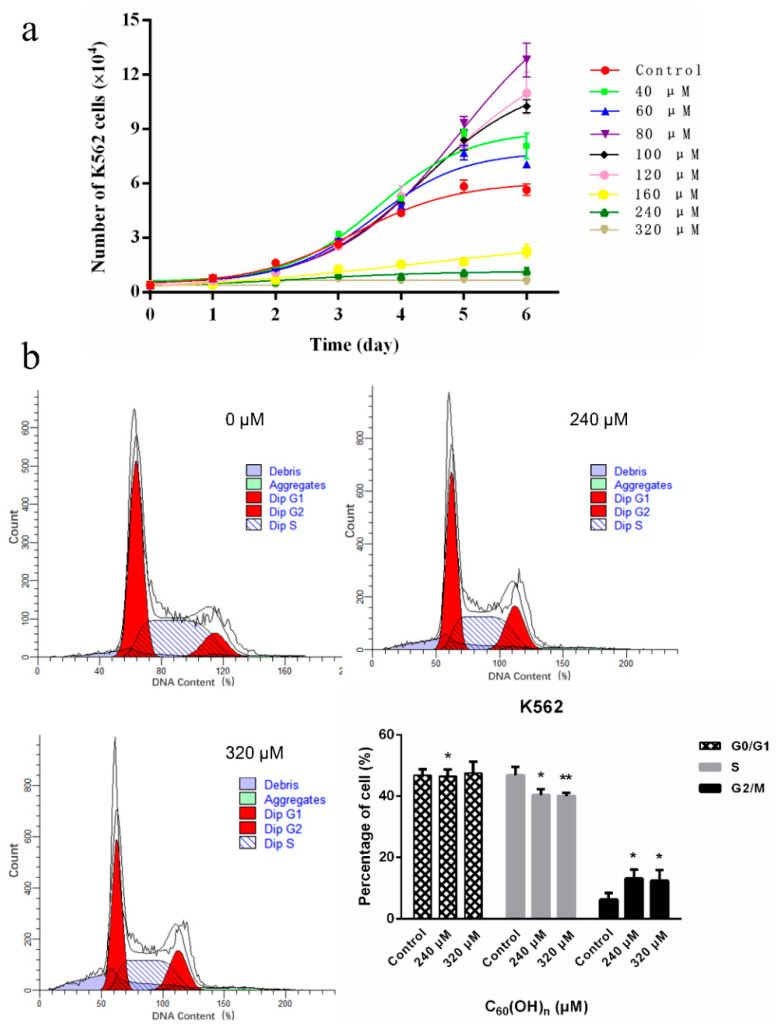
The growth curve method showed the C_60_(OH)_n_ had a dose and time-effect effect on the proliferation of cell K562 (**a**) and K562 cell cycle distribution (**b**). The (**b**) following treatment with C_60_(OH)_n_ for 48 h, K562 cells were fixed and stained with the PI solution. The first peak in the graph was G0/G1 stage, followed by a broad peak representing the S stage, and the last peak was the G2/M phase. The data are expressed as the mean ± SD of at least three independent experiments. * *p* < 0.05 and ** *p* < 0.01.

**Figure 2:**
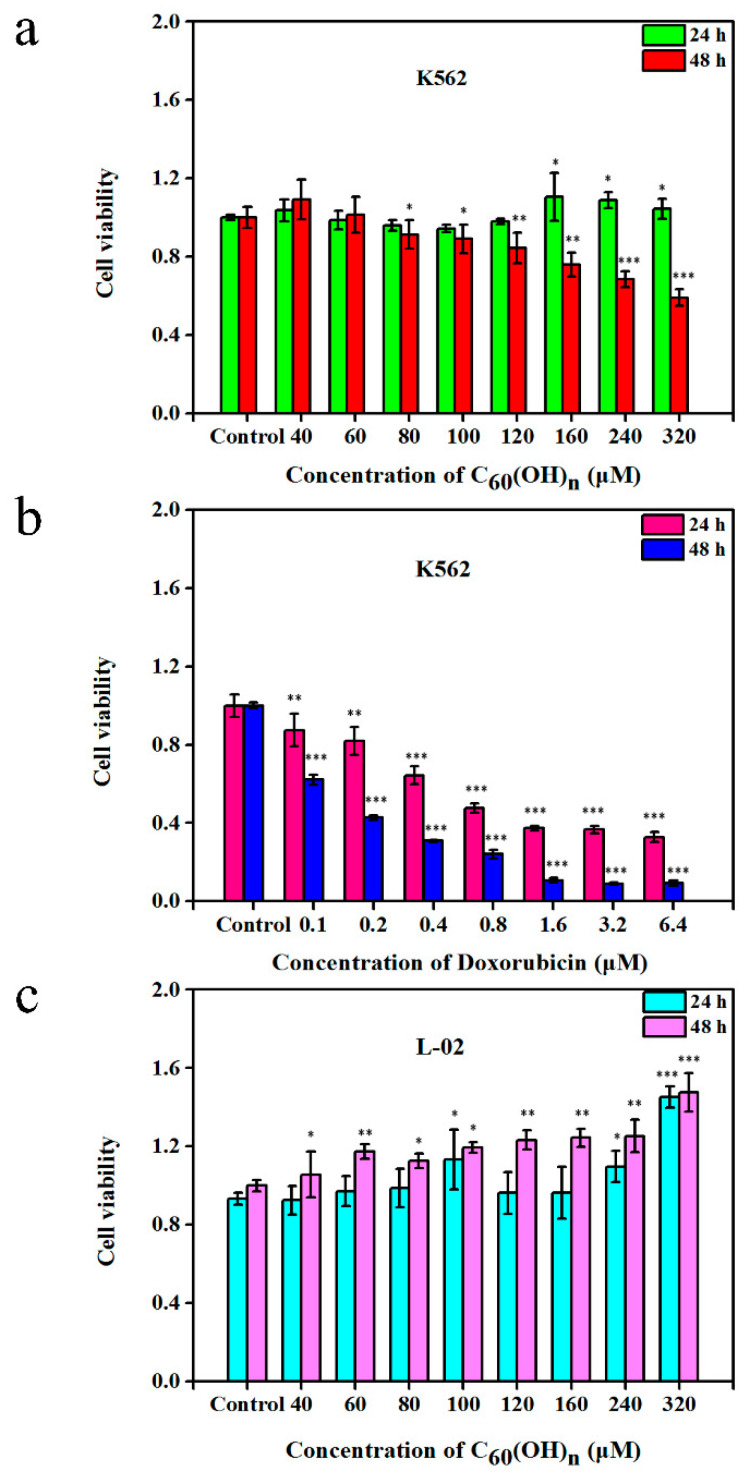
The growth-inhibitory effects of C_60_(OH)_n_ (**a**) and Doxorubicin (**b**) on human myeloid leukemia. K562 were treated with different doses of C_60_(OH)_n_ and Doxorubicin for 24 and 48 h, respectively, beside the cytotoxic effects of C_60_(OH)_n_ on normal human liver cells. L-02 cells were treated with various concentrations of C_60_(OH)_n_ for 24 and 48 h (**c**), respectively. Cell viability was assessed using a CCK-8 assay. The data are expressed as the mean ± SD of at least six independent experiments. * *p* < 0.05, ** *p* < 0.01, *** *p* < 0.001.

The result has shown that the effects of C_60_(OH)_n_ on cell proliferation have a time-dose dependent manner (Figure 1a and Figure 2). The cells were cultured in a medium containing C_60_(OH)_n_ for 24 h, and there was no obvious regular change in cell proliferation, while the cells were cultured in the same medium for 48 h and the inhibitory effect of C_60_(OH)_n_ on proliferation began to appear and was relatively stable (Figure 2). That is, when the concentration of C_60_(OH)_n_ is lower than 120 μmol/L, it can promote the proliferation of K562 cells, and when the concentration was above 160 μmol/L, the inhibition of cells began to appear. The C_60_(OH)_n_ can inhibit the growth of K562 cells significantly, with a growth inhibition rate up to 40.7% at 320 μmol/L. The results were similar to those of Chunying Chen [12], who proved that after incubation of cells with C_60_(OH)_20_ for 48 h, no significant influence on the cell viability of MCF-7 was observed at concentrations of 1, 10, and 100 μg/mL. With the increase of C_60_(OH)_n_ dose and prolonged action time, the inhibitory effect of C_60_(OH)_n_ is more obvious. Besides, the effect of C_60_(OH)_n_ on the proliferation of L-02 was studied using normal human liver cells L-02 and drug intervention as controls. The results showed that L-02 cells treated with C_60_(OH)_n_ did not show proliferation inhibitory activity, but instead promoted cell growth. Figure 1a confirms that the cell growth rate of the drug-treated group is significantly lower than that of the control group when the concentration was above 160 μmol/L. C_60_(OH)_n_ promotes cell proliferation at low concentration and inhibits its growth or induces death at high concentrations with a time-dose effect.

### 3.2. Effects of C_60_(OH)_n_ on the Cell Cycle of K562

The cell cycle of K562 cells was affected by a high concentration of C_60_(OH)_n_. It changed the ratio of S and G2-M phase cells. The S-phase cells were significantly reduced under the concentrations of 240 and 320 μmol/L, the proportion of G2-M phase cells increased significantly, and the cell cycle stagnated at the G2-M phase, with statistically significant differences (*p* < 0.05). When the concentrations of C_60_(OH)_n_ add to 640 μmol/L (not shown here), there was no significant change in the period, and the difference was not statistically significant (*p* > 0.05). It indicated that increasing concentration did not influence the cell cycle (Figure 1b).

### 3.3. Morphological Study of C_60_(OH)_n_ on K562 Cells

**Figure 3:**
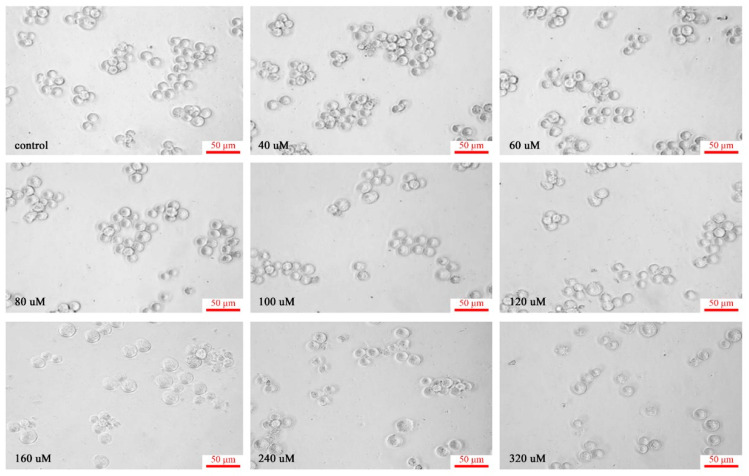
Influence of C_60_(OH)_n_ on morphological characteristic and number of human chronic myeloid leukemogenic cells K562 incubated in vitro. Morphologic changes of unstained cells caused by C_60_(OH)_n_ were measured using a fluorescence microscope (200×). Scale bars = 50 μm. The C_60_(OH)_n_ at a dosage of 40, 60, 80, 100, 120, 160, 240, 320 μmol/L, respectively, was administered to K562 cells for 48 h. All images shown were representative of three independent experiments.

K562 cells are human chronic myelogenous leukemia cell lines. Generally speaking, the cell volume is large, usually a round or oval nucleus, a large proportion of nucleus and plasma, no particles in the cytoplasm, and often with aggregation and growth [29]. However, when the cells were cultured in a medium containing different dose concentrations of C_60_(OH)_n_ for 48 h, the number of cells first increased and then decreased. The cells treated with 40 μmol/L-120 μmol/L C_60_(OH)_n_ showed little difference in morphology from the control cells. As the concentration rose above 120 μmol/L, the cells began to change; when the concentration increased to 160 μmol/L, the cell size was deformed, the connections between the cells disappeared, and the cell separated from the surrounding cells. The particles in the cytoplasm increased and thickened, and the membrane was bubbled. The number of cells floating by cell lysis also increased, and the cell morphology became more irregular. With the increase of C_60_(OH)_n_ dose, the more obvious influences on cells, and the changes in cell morphology are more prominent. When the concentration increased to 240 μmol/L and 320 μmol/L, more cells floated, and the number of cells was significantly reduced compared with that of other treatment groups, indicating that there was not only a significant inhibitory effect on cell proliferation but also a large number of cell deaths (as shown in Figure 3).

### 3.4. Effect of C_60_(OH)_n_ on Apoptosis of K562 Cells

**Figure 4:**
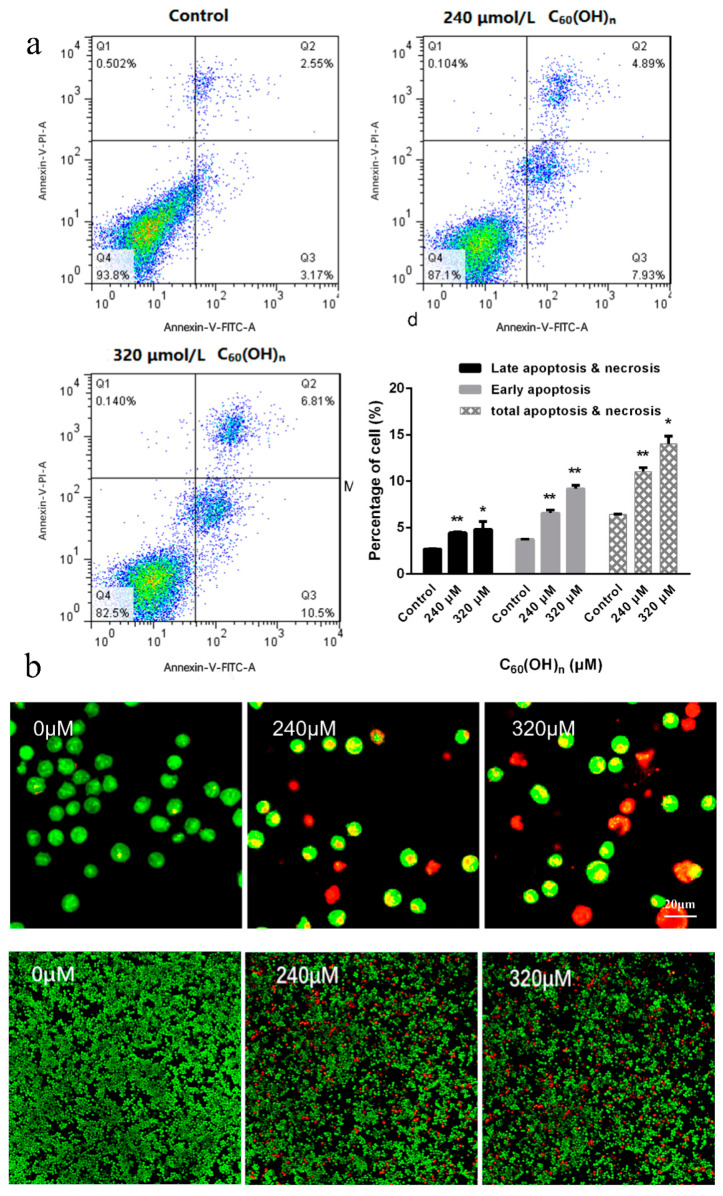
(**a**) Effects of C_60_(OH)_n_ on the induction of apoptosis. K562 cells were treated with C_60_(OH)_n_ for 48 h, and the apoptotic cell rate was analyzed using Annexin-V-FITC/PI staining. Data were presented as the mean ± SD of three independent experiments. * *p* < 0.05 and ** *p* < 0.01. (**b**) Representative images of nuclear condensation and DNA fragmentation in K562 cells exposed to C_60_(OH)_n_ (600× and 100×). Nuclei were detected by AO/EB co-staining.

As a result of the greater inhibition effect of fullerene on K562 cells at the concentrations of 240 μmol/L and 320 μmol/L, the two concentrations were selected for further investigation. The apoptotic K562 cells rate was analyzed by using Annexin V-fluorescein isothiocyanate (FITC)/propidium iodide staining and flow cytometry after the action of different concentrations of fullerenol on leukemia cells for 48 h. Comparing with other detection methods based on nuclear changes, the early stages of apoptosis can be identified more effectively. Annexin V staining represents a loss of membrane integrity, which can also occur in the anaphase of cell death. Therefore, Annexin V-FITC/PI staining is often used to identify the integrity of cell membranes, and a combination of positive/negative signals was used to differentiate early or late apoptosis and necrosis [30]. As shown in Figure 4a, C_60_(OH)_n_ (n = 18–22) could accelerate both early apoptosis and late apoptosis in K562 cells, the 320 μmol/L groups were more obvious than 240 μmol/L groups. It was also found that the proportion of apoptotic cells increased significantly after the treatment of high concentrations of C_60_(OH)_n_ (240, 320 μM) for 48 h compared with the negative control group (*p* < 0.05). These results are consistent with the morphological experiments.

No apoptotic cells were observed under a fluorescence microscope in the normal control group. Early apoptotic cells were observed in the 240 μmol/L groups after 48 h C_60_(OH)_n_ (n = 18–22) treatment. Specifically, after AO/EB staining, the nuclei were observed to be yellow-green fluorescent, granular, and clustered on one side of the cell, showing the budding of the cell. The number of early apoptotic cells and the number of late apoptotic cells were positively correlated with the concentration. The latter is characterized by nuclear concentration and migration, and apoptotic bodies are seen (Figure 4b), apoptotic bodies being one of the important markers of cell apoptosis [31]. 

### 3.5. Effects of C_60_(OH)_n_ on Mitochondrial Membrane Potential in K562 Cells

The changes of mitochondrial membrane potential in leukemia K562 cells treated with C_60_(OH)_n_ were analyzed by multi-parameter flow cytometry using the fluorescent probe JC-1. In the process of cell apoptosis, the mitochondrial membrane structure of the cell damaged, the membrane potential was reduced, the concentration decreased, and JC-1 could not be concentrated in the mitochondrial matrix—mainly in the form of monomers—so that the red fluorescence intensity produced by the apoptotic cells was reduced. When excited at 488 nm, the maximum emission wavelength was 527 nm, showing green fluorescence, which was detected by FL1. When the membrane potential is high, the concentration is high, and aggregation is formed. When the 488 excitation occurs, the maximum emission wavelength is 590 nm, showing red fluorescence, FL2 was used for detection, and FL2 was used for the scatter plot of FL1.

**Figure 5:**
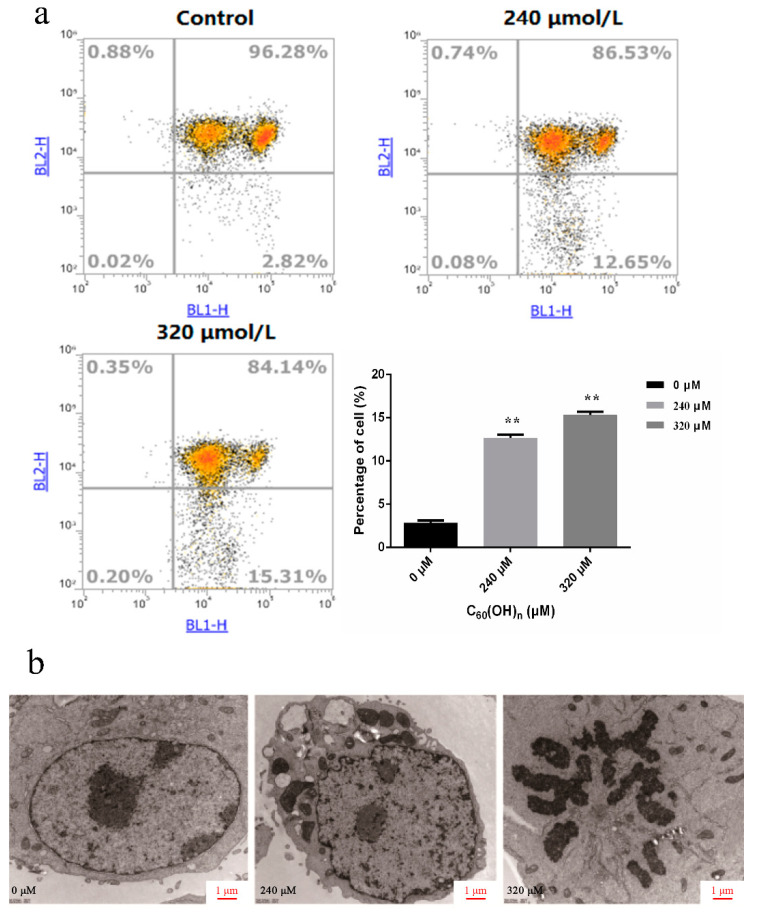
(**a**) Flow cytometry analysis of MMP (ΔΨm) based on JC-1 staining. Cells were cultured with C_60_(OH)_n_ for 48 h and stained with JC-1. ** *p* < 0.01. (**b**) Observation of characteristic ultrastructural changes in K562 cell apoptosis after treatment with C_60_(OH)_n_(n = 18–22). The ultrastructure of the apoptosis in K562 cells following treatment with 240, 320 μmol/L C_60_(OH)_n_ for 48 h, respectively, was observed under the electron microscope. All images shown were representative of three independent experiments that appeared as similar results. Scale bars = 1 μm.

As shown in Figure 5a, cells in the control groups had higher activity and higher mitochondrial membrane potential. The concentration of JC-1 aggregates in mitochondria was high, and the red fluorescence was strong. The cells were concentrated in the first quadrant, and the cells in the fourth quadrant were few, accounting for only 2.82%. In 240 and 320 μmol/L treated cells, the number of cells in the fourth quadrant increased gradually, accounting for 12.65% and 15.31% of the total number of cells, respectively. These results suggested that the increased dose of C_60_(OH)n (n = 18–22) caused a significant amount of cell apoptosis. In the fourth quadrant, the cell red light decreased, in which the concentration of JC-1 aggregation decreased. This was due to the apoptosis induced by C_60_(OH)n, resulting in the decline of mitochondrial membrane potential, and the concentration of JC-1 in mitochondria decreased accordingly. This indicated that C_60_(OH)n induced apoptosis through the signal transduction pathway of mitochondrial cell apoptosis.

### 3.6. Ultrastructural Changes of Cell Apoptosis

The general characteristics of the ultrastructure of leukemia K562 cells were observed under transmission electron microscopy: large cell size, clear intercellular space, weak cell connection, intact cell membrane and nuclear membrane, double layer unit membrane, few organelles, complete mitochondrial ridge, and abundant mitochondria. There are large amounts of endoplasmic reticulum in the cytoplasm, mitochondria and rough endoplasmic reticulum are irregular and disorganized. With the increase of C_60_(OH)_n_ (n = 18–22) concentration, the apoptotic K562 cells gradually increased and became more obvious. Some of the cells showed typical apoptotic cell morphological changes: the morphology became irregular, the cytoplasmic hollow vesicles increased, the chromatin in the nucleus agglutination, edge aggregation, etc. In the control group of 320 μmol/L drugs, the proportion of cytoplasmic concentration increased, the nuclear membrane broke and disappeared, the chromatin was dispersed into several small pieces, and the chromatins—such as mitochondria—were solid and shrunken. The nucleus was fragmented, but the cell membrane was intact and apoptotic bodies were visible (Figure 5b).

It was seen that high doses of C_60_(OH)_n_ induce the K562 cell death in a certain way and the characteristic morphological changes were observed by transmission electron microscopy, that is, the proportion of nucleoplasm increased, and the nuclear material was dense, patchy, or gathered in the nuclear membrane in a crescent shape, appeared the apoptotic bodies.

## 4. Discussion

Due to its superior antioxidant characteristics, fullerene has been rapidly developed as a new nanomaterial in the field of biomedicine. Fullerenol is a fullerene derivative. Due to the increased number of hydroxyl groups on the surface of fullerenol, it is more antioxidant than fullerene. Its impact on tumor cells is based on the elimination of excessive free radicals. There is mounting evidence that high levels of free radicals can result in oxidative stress and metabolic dysfunction, which can result in a range of diseases, including cancer [21]. Due to the high concentration of hydroxyl groups in fullerenols, they are more likely to form hydrogen bonds with free radicals, resulting in the formation of collagen protein macromolecules attached to tumor cells, stimulating autophagy, and promoting the occurrence and metastasis of tumors, inducing apoptosis [32,33]. Fullerenol C_60_(OH)_24_ has been shown in previous research to inhibit H_2_O_2_-induced apoptosis in A549 cells by boosting Nrf-2-mediated redox state and enzyme activity [34]. C_60_(OH)_n_ (n = 6–24) exhibits a substantial inhibitory effect on the proliferation of human breast cancer cells (T47D and MCF-7 cells). There is no clear toxicity to vital organs and no statistically significant difference between the CDDP, doxorubicin, and paclitaxel concentrations [6,35]. The C_60_(OH)_20_ nanoparticles can efficiently assist the immune system in eliminating tumor cells, ultimately impairing tumor tissue viability through TNF-α mediated cellular immunity [36]. However, the specific molecular mechanism of fullerenol affecting leukemia K562 cells remains to be studied.

The results obtained from this study imply that C_60_(OH)_n_(n = 18–22) promoted proliferation at low concentrations (less than 120 μmol/L), but inhibited cell proliferation and led to cell apoptosis in high doses (160–320 μmol/L). We further explored the morphological change and ultrastructural characteristics of K562 cells, wherein obvious morphological changes and the appearance of apoptotic bodies were observed. We found that C_60_(OH)_n_ (n = 18–22) can prevent K562 cells from entering the G2-phase, resulting in a decrease in mitochondrial membrane potential and cell death. These results supported that fullerenol C_60_(OH)_n_ at high concentrations induces apoptosis in chronic myeloid leukemia K562 cells and shows a good dose-effect relationship. However, at low concentrations there was no cytotoxicity or harm to L-02 and K562 cells, and it even stimulated their proliferation. Simultaneously, this finding is compatible with several studies indicating that fullerenes have no hazardous side effects. In addition, we discovered that the fullerenol treatment group’s mitochondrial membrane potential was much lower, implying that K562 cell death is linked to the signal transduction pathway of mitochondria-induced cell apoptosis. The mechanism of high concentration fullerenol inhibiting the proliferation and inducing apoptosis of K562 cells may involve a complex process of multiple signal transduction pathways, and apoptosis may be one of the mechanisms of its anti-tumor effect. In conclusion, fullerenols of high concentration may cause cell death in various ways including apoptosis, autophagy, and macromolecule formation, which may be caused by a series of damages produced by high concentration, rather than the drug’s inhibitory action on K562 cells.

Current cancer treatments mainly include changing the microenvironment, anti-angiogenesis, drug resistance, improving immunity, mediated chemical sensitization, and so on, but most of the fullerenols antitumor research has focused on breast cancer, lung cancer, and other solid tumors. Since leukemia is a non-solid tumor, the internal environment cannot be controlled, and the effect should act directly on the cancer cells themselves. Thus, the synergistic effect of fullerenol and commonly used chemoradiotherapy drugs can be considered for solid tumors, which can not only inhibit tumors but also reduce the toxicity and side effects brought by chemoradiotherapy. The use of antioxidants as co-adjuvant therapy can reduce the toxic and side effects of pro-oxidant drugs, while it can reduce the cytotoxicity of many drugs. However, our experimental results do not show a therapeutic effect of C_60_(OH)_n_ for chronic myeloid leukemia of K562 cells. In future studies, we endeavor to change the number of hydroxyl groups, add amino acids or functional modification functions on fullerenols to explore their influence on leukemia cells and animal research. It could also provide a basis for further research on the therapeutic effects of fullerene derivatives on non-solid tumors, such as leukemia.

## Data Availability

The data sets used and analyzed that support the findings of this study are available from the corresponding author upon reasonable request.
